# The Influence of Organic Material and Temperature on the Burial Tolerance of the Blue Mussel, *Mytilus edulis*: Considerations for the Management of Marine Aggregate Dredging

**DOI:** 10.1371/journal.pone.0147534

**Published:** 2016-01-25

**Authors:** Richard S. Cottrell, Kenny D. Black, Zoë L. Hutchison, Kim S. Last

**Affiliations:** 1 Scottish Association for Marine Science, Oban, Argyll, Scotland, PA37 1QA; 2 School of Biology, University of St Andrews, St Andrews, Fife, KY16 9ST, Scotland; Bangor University, UNITED KINGDOM

## Abstract

**Rationale and Experimental Approach:**

Aggregate dredging is a growing source of anthropogenic disturbance in coastal UK waters and has the potential to impact marine systems through the smothering of benthic fauna with organically loaded screening discards. This study investigates the tolerance of the blue mussel, *Mytilus edulis* to such episodic smothering events using a multi-factorial design, including organic matter concentration, temperature, sediment fraction size and duration of burial as important predictor variables.

**Results and Discussion:**

Mussel mortality was significantly higher in organically loaded burials when compared to control sediments after just 2 days. Particularly, *M*. *edulis* specimens under burial in fine sediment with high (1%) concentrations of organic matter experienced a significantly higher mortality rate (p<0.01) than those under coarse control aggregates. Additionally, mussels exposed to the summer maximum temperature treatment (20°C) exhibited significantly increased mortality (p<0.01) compared to those in the ambient treatment group (15°C). Total Oxygen Uptake rates of experimental aggregates were greatest (112.7 mmol m^-2^ day^-1^) with 1% organic loadings in coarse sediment at 20°C. Elevated oxygen flux rates in porous coarse sediments are likely to be a function of increased vertical migration of anaerobically liberated sulphides to the sediment-water interface. However, survival of *M*. *edulis* under bacterial mats of *Beggiatoa spp*. indicates the species’ resilience to sulphides and so we propose that the presence of reactive organic matter within the burial medium may facilitate bacterial growth and increase mortality through pathogenic infection. This may be exacerbated under the stable interstitial conditions in fine sediment and increased bacterial metabolism under high temperatures. Furthermore, increased temperature may impose metabolic demands upon the mussel that cannot be met during burial-induced anaerobiosis.

**Summary:**

Lack of consideration for the role of organic matter and temperature during sedimentation events may lead to an overestimation of the tolerance of benthic species to smothering from dredged material.

## Introduction

In order to meet demands for construction, beach nourishment and land reclamation, aggregate dredging is becoming an increasingly significant industrial pressure upon the marine environment [1–4]. Consequently there is a growing need to understand the environmental risk posed by such activity in order to minimise negative impacts upon marine systems [4–6].

The direct impacts of dredging on the physical and biological condition of the seafloor are well documented. Dredging operations lift gravel and sand from the seafloor, before being transferred to a hopper for screening [7]. The direct physical effects of substratum removal include the alteration of bottom topography and flow regime, increased turbidity and the shift to a finer sediment fraction composition due to the settlement and subsequent resuspension of screening fall-out [8–11]. The alteration to sediment characteristics may favour recolonisation by opportunistic species, yielding a different community structure to that of pre-disturbance conditions [8,12]. Furthermore, biological impacts can include the direct removal of infaunal and epifaunal species and potentially disrupt larval settlement through removal of suitable substrate [8], impacting both species richness and biomass of benthic communities [12].

In contrast, less attention has been paid to the extent of the initial biological impacts induced by the settlement and resuspension of overspill and rejected material from aggregate screening processes. Fine sediment fractions amounting to between 20–80% of dredged sediment material [7,12,13], less favourable in aggregate acquisition, are released from dredging barges using reject chutes. Discharged material creates a plume of highly concentrated Suspended Particulate Matter (SPM) leading to heavy sedimentation within a few hundred metres of dredging activity [8,13,14]. Resuspension of these finer particles may increase the impact zone of dredging activities to several kilometres downstream of the original perturbation [12]. Ultimately heavy and episodic deposition or ‘over-sanding’ events such as these may have considerable implications for benthic communities–especially those residing in coarser, gravel environments that are unaccustomed to such dramatic fluctuations in SPM and sediment deposits [15,16]. While population responses are relatively well reported [8,15,17], there is a paucity of studies regarding individual species response in terms of behaviour and survival.

In response, Hendrick *et al*. [16], designed a range of experiments aimed at quantifying the effects of smothering on prominent benthic species from UK waters commonly associated with dredged environments. Hutchison et al., [18] then studied the behavioural response of the blue mussel, *Mytilus edulis* which displayed moderate tolerance to burial events. Limited escape behaviour was exhibited at burial depths of 2 cm but no deeper [18], indicating the species’ vulnerability to heavy sedimentation events. Additionally *M*. *edulis* displayed a greater mortality with increased duration of burial and with decreased sediment fraction size, which is highly significant to populations affected by screening fallout [8,14]. While these two studies were invaluable for studying the physical effects of smothering, the commercially obtained sediment was free of organic material to minimise any changes to sediment biochemistry. In reality, discharged sediment from dredgers will be loaded with organic matter (OM) [12] as a result of homogenising benthic biota and detritus during substratum removal [19].

Effects of organic enrichment on benthic populations has been robustly studied in regards to aquaculture waste deposition [20–26] and although relatively scarce in marine sediments, OM reactivity is highly influential upon chemical and therefore biological conditions within the benthic environment [27,28]. Both aerobic and anaerobic mineralisation of OM has implications for oxygen availability and the production of noxious metabolites which may be highly poisonous [27]. Therefore, the presence of labile organic material within sediment is likely to have great consequence for the survival of fauna buried within it.

Since penetration of oxygen into marine sediments is limited by the Diffusive Boundary Layer (DBL), oxic metabolism of OM may be of negligible significance beyond a few millimetres below the sediment surface. Instead, the abundance of sulphate [SO_4_^2-^] in seawater means that sulphate reduction is often the most dominant anaerobic process in coastal sediments [29,30]. This is of significance to most benthic fauna as sulphides can exert toxicity through their ability to penetrate biological membranes and interrupt aerobic respiratory processes through the inhibition of cytochrome *c* oxidase [31–34]. Therefore, the burial of benthic fauna in organic loaded sediment has the potential for profound effects on mortality of specific species through sulphide exposure [32,35–37].

Furthermore the quality and quantity of OM in sediment may be influential in affecting nutrient availability for pathogenic bacterial communities. Availability of dissolved organic material in marine sediment is known to be the limiting factor for growth of heterotrophic bacteria [38,39] and its presence plays an important role on aiding infection in a variety of marine species [38,40,41]. Notably, a number of studies have revealed a greater susceptibility to infection under anoxic conditions in many different bivalve species, citing bacterial propagation as the casual factor for mortality under anaerobiosis [42–45]. Consequently it is likely that nutrient enriched, highly reducing burial sediments that yield an ideal environment for bacterial proliferation, would reduce survival time of benthic fauna under burial compared with those in an organically poor burial medium.

This study aims to extend previous works [16] to specifically address the role of organic material on the burial tolerance of *M*. *edulis*–a prominent intertidal and sub-tidal species well recognised as ecosystem engineers and of overt commercial importance. These mussels can form extensive beds in temperate waters, increasing habitat complexity, heightening local biodiversity and significantly contributing to benthic-pelagic coupling [46–48]. The extended range and depth distribution of *M*. *edulis* in coastal water around the UK [49–51], one of the world’s largest extractors of marine aggregates, [1,4,15,52,53] makes it a particularly appropriate species to study the effects of smothering from dredging outwash. The burial tolerance of *M*. *edulis* has been previously considered as moderate according to theoretical sensitivity assessments [53] and this was in close agreement with Hutchison *et al*. [18]. However the role of reactive OM contained within burial sediments has not been considered until now.

Consequently it is hypothesised that increasing concentrations of labile organic material within the sediment will lead to a reduction in survival time of *M*. *edulis* under burial (>2 cm). Tolerance of *M*. *edulis* was assessed using a multi-factorial experimental design incorporating temperature, sediment fraction size, duration of burial and OM concentration. It was expected that mortality would be increased under higher temperatures, finer aggregate fractions, increased burial duration and higher organic load.

## Materials and Methods

### 2.1. Experimental Organisms

*M*. *edulis* specimens used in this study were sourced live from local line culture on 13 June 2013 from J MacGregor and Sons Ltd, Isle of Seil, Argyll, Scotland. Mussels were allowed a 48 h recovery and feeding period after aerial exposure, achieved through suspension in creels from the pontoon at the Scottish Marine Institute. All mussels were then transferred to seawater flow-through holding tanks to acclimatise to experimental temperatures for sixteen days [54]. Specimens selected for experimental use were cleaned of loose material around their shell and controlled for size (mean 53.4 mm ± 7.3), with individuals of extreme size or with live fouling omitted to reduce the influence of confounding factors.

### 2.2. Ethics Statement

All experiments conducted complied with current laws regarding animal welfare in the UK. While the inclusion of live models was necessary for this study, only the required number of healthy mussels were segregated at an early stage from stock to reduce number of individuals exposed to experimental conditions thus minimising density dependent mortality through crushing or metabolic stress.

### 2.3. VOrtex Resuspension Tanks

All experimental burials were conducted with 12 VOrtex Resuspension Tanks (VORTs) as described in [16,55]. Each VORT was supplied with sub-sand filtered seawater at a rate of 50–60 L h^-1^ (flow meters supplied by GEMO) and maintained at 200 L volume. All tanks were temperature controlled using 1kW aquarium heaters and sensors (supplied by AquaMedic Commercial Ltd). Six of twelve tanks were heated to 20°C representing UK summer maximum temperatures and the remaining six ambient tanks were heated to 14°C creating a minimum control.

The VORTs are designed to create a turbulent unidirectional water current using Oceanrunner pumps (supplied by AquaMedic Ltd). Acoustic Doppler Velocimetry (ADV) (supplied by Nortek Ltd) calculated mean current velocity to be 2.84 cm s^-1^ (+1.46) on the outside and 1.04 cm s^-1^ on the inside of the VORT. While maximum current velocities at dredge sites may be significantly higher than this [15,56], it provided a more realistic set up than static conditions inside the VORTs. Airlift systems brought water from the VORT base back to the surface through a central column, maintaining oxygen saturation of seawater. Overhead lighting was controlled via time-switch, creating a 12:12 hour light: dark cycle to match ambient photoperiods during the study period.

### 2.4. Experimental Aggregates

Two fraction sizes of commercially obtained sediment (supplied by Specialist Aggregate Ltd) were used for experimental burials–coarse (described as 1.2–2.0 mm by manufacturer) and fine (0.1–0.3 mm). Laser particle analysis by Hendrick *et al* confirmed described fraction sizes of experimental aggregates to be reasonable [16]. Organic content of experimental sediments were quantified using Loss on Ignition (LOI) analysis following protocol outlined by Asknes *et al* [57]. Samples (2 g) were oven dried at 95°C for 6 h and subsequently combusted at 500°C for 6 h. Ash free dried weight lost during ignition was then determined:

$$TOMC=\left[\left(DW_{95}-DW_{500}\right)/DW_{95}\right]\times 100$$ Eq 1

Where *TOMC* = Total Organic Matter Content, *DW*_95_ = dry weight of sediment sample after 6 h at 95°C, *DW*_500_ = dry weight of sediment sample after 6 h at 500°C.

Porosity of each sediment fraction was also determined by comparing saturated and dry weight sediment samples following methodology described by Avnimelech *et al*. [58]:

$$V_{t}=V_{s}+V_{w}$$ Eq 2

$$p=\left(V_{w}/V_{t}\right)\times 100$$ Eq 3

Where V_t_ = total volume of sediment sample, V_s_ = weight of dry sample (g)/ sediment particle density (taken to be 2.65 g cm^-3^ for inorganic sediments), V_w_ = weight of wet sample (g)–weight of dry sample (g), p = porosity of sediment.

### 2.5. Addition of Organic Material

Simulating highly reactive biotic detritus expected in dredging fallout was achieved through the integration of fish feed pellets supplied by BioMar Ltd. Previous elemental combustion analyses of the pellets revealed a C:N ratio of 8.78 (± 0.59) [59].

To reduce heterogeneity of OM added to sediment and to further simulate homogenised organic slurry present in dredging discards [12,19], pellets were ground using a food processor and further homogenised for 2 minutes at 330 rpm using an agate planetary ball grinder (Retsch PM 400). Pellet weight needed for each treatment was then mixed into sediments by alternately exchanging the mix between two buckets until an even color of mix was achieved, usually after only a few exchanges. To ensure complete mixing and standardise between samples (in total ~240 kg of sediment was used during the study) 30 exchanges were carried out of the sediment-OM mix.

Organic content of finely ground pellets was quantified using LOI analysis protocol detailed in section 2.3. LOI analysis results were used to calculate un-dried pellet weight needed to create proportional organic content of experimental sediments for control sediments (0% added), 0.1% and 1% added OM treatments. Organic content in hopper overspill is known to be highly variable and may equate to over 10% of dried discard samples [19]. However, considering the reactivity of fish pellets, OM concentrations were kept low within the likely in-situ range and differed by an order of magnitude to create a scale for observation.

### 2.6. Sediment Total Oxygen Uptake

Sediment Total Oxygen Uptake (TOU) rates were determined as a proxy for the mineralisation of organic material in the sediment in order to support the burial tolerance data through an understanding of biochemical processes within burial sediments. Ground fish feed pellets were integrated into 50 g sediment samples to simulate the two experimental OM treatments from burial experiments (0.1 and 1% added OM). None was added to sediment controls. Samples were contained within 200 ml water tight respiration chambers topped with unfiltered seawater stirred constantly by a magnetic flea and subsequently incubated at either ambient or summer maximum temperatures within water baths.

Dissolved Oxygen (DO) concentrations (mg/L) were measured non-invasively at a rate of 1 reading per second (Presens Fibox 3 Fibre Optic Meter with planar oxygen-sensitive foils) with temperature compensation (range: 0–250% air saturation and 0–50°C; accuracy: ± 1% air saturation at 100% & ± 1°C respectively). All incubations were kept in the dark with measurements taken every two hours between 8 am–8 pm for logistical reasons. The depth of the water column overlying the sediment in the 200 ml chambers ranged between 80.6–86.1 mm with a diameter of 51.5 mm. Overnight data gaps were modelled under the assumption that the rate of oxygen decrease in a well-mixed overlying water column is approximately linear [27]. Linear model slope and intercept parameters were optimised using Microsoft Excel’s solver function to minimise the sum of squared residuals (Ʃ(O-E)^2^) between modelled and measured data values. Interpolated data was then calculated using the standard linear equation (y = mx+c, where m = slope coefficient, x = time (days) and c = intercept). Slope parameter estimates were then used to calculate TOU rates (mmol m^-2^ day^-1^) using the following protocol [27]:

TOU=(m/32)×(V/A) Eq 4

Where m = slope estimate for DO depletion curve (mg/L day^-1^), V = volume of water column (L), A = surface area of sediment sample (m^2^).

### 2.7. Burial protocol

The burial protocol followed a multi-factorial design with minor adaptations to that of Hendrick *et al*. [16]. Experimental variables, included OM content, temperature, sediment fraction size and burial duration (logged to account for uneven time steps in duration treatments). Size of mussel, VORT number and position in VORT were monitored variables included in initial model fit to be able to exclude their influence. *M*. *edulis* specimens (n = 180) were randomly assigned to different burial treatments in PVC pots with an internal diameter of 76 mm and capped at the base by a plastic dish. The top of the burial chamber was exposed to the water column and each specimen was buried in either coarse or fine sediment with 0, 0.1 or 1% added OM loadings for 2, 4, 8, 16 or 32 days at either ambient (mean 15.38°C, range 2.01°C) or summer maximum treatments (mean 19.98°C, range 0.16°C). See S1 Table for full details of burial treatments. Burial depth remained constant at 5 cm (from the top of the mussel) to represent moderate sediment overburdens expected within the immediate vicinity (< 500 m) of dredging activities [14]. All burial pots were randomised within their temperature treatment between VORTs, yielding a mean of 25 pots per VORT. Two types of unburied control treatments (n = 120, see S2 Table) were randomly allocated between temperature treatments to the VORTs. *M*. *edulis* specimens in burial chambers without burial were added to control for any pot effect and individuals without chambers were placed on the mesh base of the VORT to control for any VORT effect.

Prior to burial, mussels were cleaned of organic debris including byssal threads and subsequently measured using callipers along their longest axis. The position of each burial or control specimen within the VORT was also recorded (inside or outside). Salinity and pH was measured daily in all VorRTs during the 32 day burial period (July 01 –August 03 2013) using a Wissenshaftelich-Technische WerkStatten (WTW) Multi 340i Universal Pocket Meter with temperature adjusted dipping probes. Two-point calibration for pH and conductivity was achieved through pH 2.00, 4.01, 7.00 and 10.01 (at 25°C) buffer solutions and conductivity standards (0.01 mol l^-1^ potassium chloride). DO saturation and temperature of VORT water was also monitored daily using the Fibox 3 with dipping probe throughout the study period.

### 2.8. Mortality Assessment

Upon termination of the burial period for each treatment, *M*. *edulis* specimens were removed from the sediment and mortality was assessed. Mortality was indicated by shell gape and was confirmed if gape persisted after 1 minute of regular mantle contact with tweezers known to result in a shell closure reflex response. Any unresponsive individuals were transferred to aerated flow through tanks for 24 h, after which mortality was confirmed. In the instance that the tissue mass had fully decomposed and come free from the shell, the mortality assessment was considered unnecessary.

### 2.9. Statistical Analyses

Cross tabulation and Chi-Squared Analyses on burial data were conducted in Microsoft Excel 2007. All further exploratory data and statistical analysis were conducted using R statistical package version 2.15.3. Shapiro-Wilk tests were used to assess normality of data needed for parametric test assumptions. Two-way Analysis of Variance (ANOVA) and post-hoc Tukey’s Honest Significant Difference (HSD) tests were used to compare TOU rates between fine and coarse control sediments at both temperatures. One-way ANOVA and post hoc testing was applied to all results within the LOI and porosity analyses. Generalised Linear Models (GLMs) from binomial and Gaussian distribution families were fitted to mortality and all TOU data respectively using all-possible-subset model selection techniques. TOU data was logged to account for over-dispersion and relative model fit was determined using Corrected Akaike Information Criterion (AICc) scores to allow comparison between models with finite sample sizes [60,61]. In contrast, the binary nature of mortality data from burial experiments meant that absolute model fit was determined using:

$${\text{R}}^{2}{=(1\text{-exp}((\text{D-D}}_{\text{null}}{)/\text{n}))/(1\text{-exp}(\text{-D}}_{\text{null}}/\text{n}))$$ Eq 5

Where D = deviance of the fitted model, D_null_ = deviance of the intercept only model [62]

Calculating probability of mortality on the scale of the response from binomial model output was achieved through using the inverse of the logit link function:

$$p_{i}=\exp^{\beta x+\beta 1x1+\beta nxn}/(1+\exp^{\beta 0+\beta 1x1+\beta nxn})$$ Eq 6

*Where* p_i_ = probability of mortality under the *i*^*th*^ burial treatment, β_x_ = coefficient for predictor variable x, x = value of predictor variable, n = *n*^*th*^ number of variables

The difference in probabilities derived from exponentiation of model coefficients were then tested for significance using a hypothesis Z-test.

## Results

### 3.1. Sediment Analysis

LOI analysis of sediments prior to OM addition revealed 0.293% (± 0.0818) OM content in coarse sediment and 0.06% (± 0.0149) OM content in fine sediments. One-way ANOVA yielded significant differences (F 7.70, df = 1, p = 0.0501) between the organic contents of the sediment fractions. One-way ANOVA and post-hoc analysis showed coarse sediment to have significantly greater porosity than the fine fraction (F = 27.18, df = 1, p<0.01).

Fish feed pellets yielded 10.2% water content upon drying and LOI analysis of dried ground dry pellets revealed 90.6% OM content, implying 81.4% total organic content of un-dried weight. This value was then used to calculate pellet quantities needed for each sediment treatment.

### 3.2. Total Oxygen Uptake of Experimental Sediments

**Fig 1:**
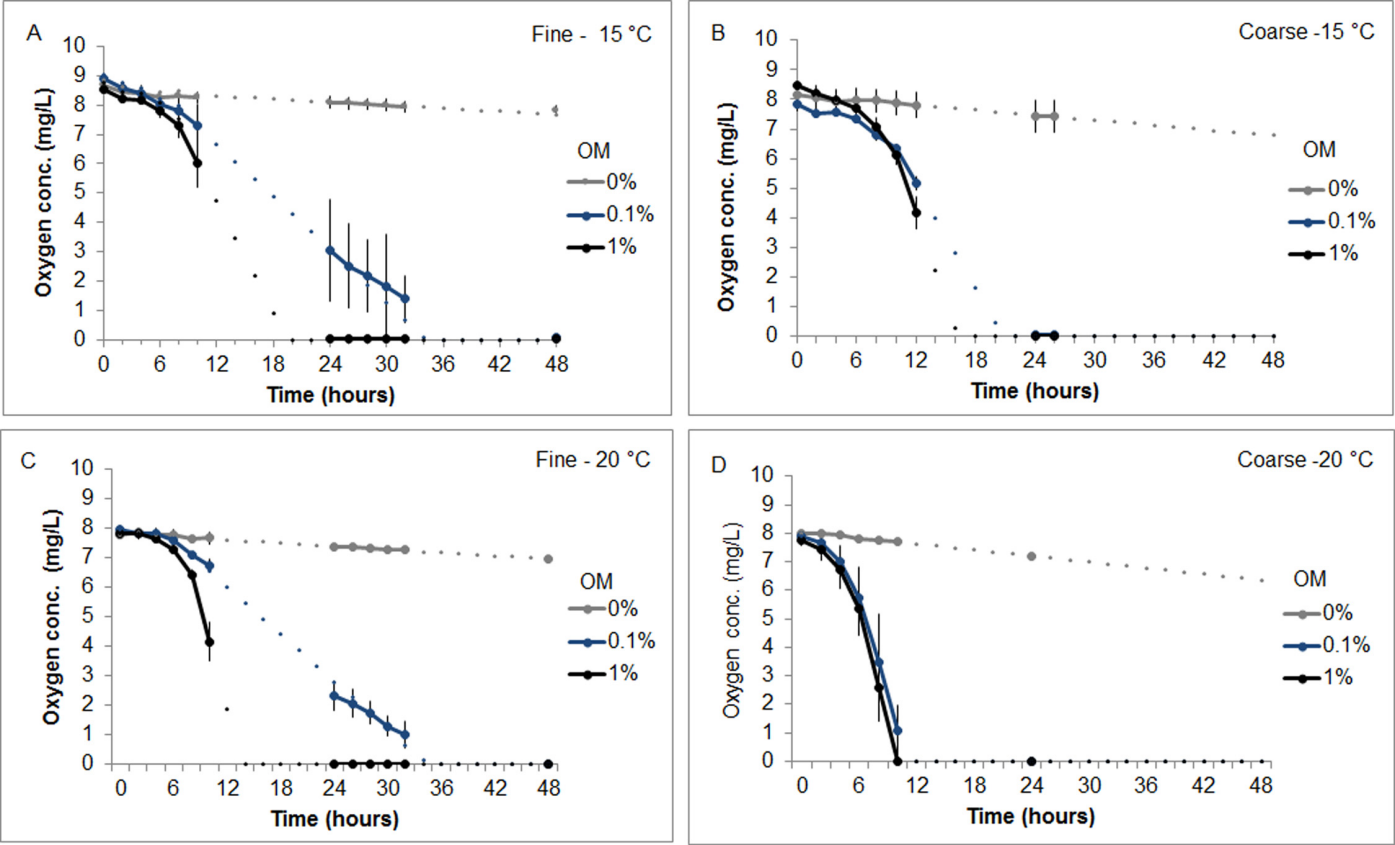
Oxygen Depletion Curves from Sediment Metabolism Incubations. Depletion curves display mean oxygen concentration values for triplicate samples of each OM treatment (0, 0.1 or 1%) at their respective temperatures. Error bars represent standard error of replicate oxygen concentrations for each time step. Dotted lines between solid lines represent best fit inter/extrapolations. **A.** Oxygen depletion curve for fine sediment at 15°C. **B.** Oxygen depletion curve for coarse sediment at 15°C. **C.** Oxygen depletion curve for fine sediment at 20°C. **D.** Oxygen depletion curve for coarse sediment at 20°C.

Temperatures were well maintained in both ambient (15.2°C ± 0.0116) and summer maximum (19.9°C ± 0.0122) TOU incubation experiments. Fibre-optic measurements of oxygen concentrations were averaged between triplicate chambers of each treatment to create oxygen depletion curves. Model fit for linear interpolations between data readings were conducted for each chamber and are displayed by dot points in Fig 1. Variation in depletion gradients between chambers is indicated by the data point error bars.

**Fig 2:**
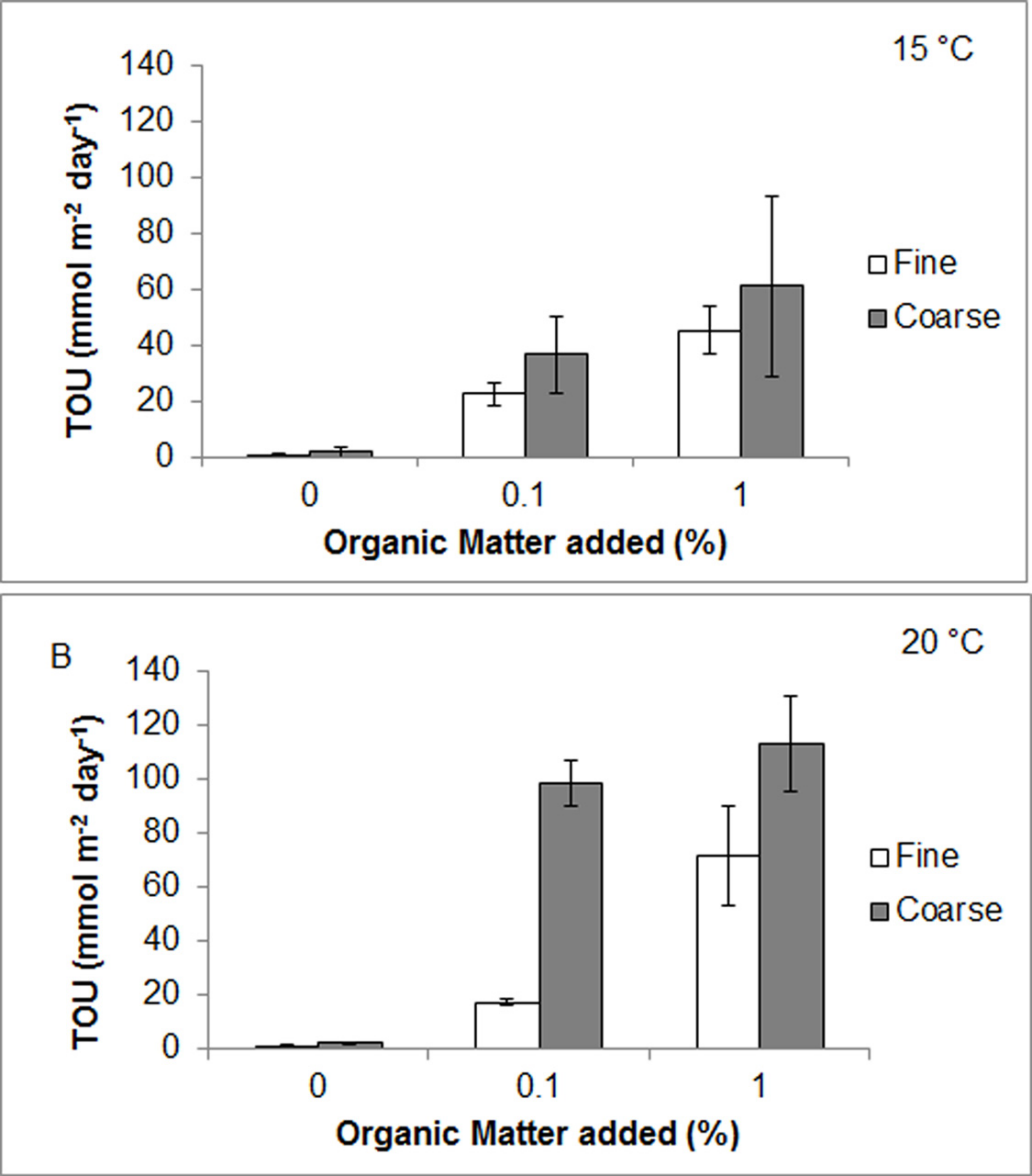
Experimental Sediment Total Oxygen Uptake (TOU) rates. Oxygen flux rates during closed incubations for experimental sediments under varying organic matter treatments (0, 0.1 and 1%) at **A** ambient (15°C) and **B** summer maximum temperatures (20°C). Error bars represent standard error.

Oxygen depletion was greatest with 1% organic loadings in the summer maximum temperature treatment and with coarser sediments. Notably pooled TOU rates were also higher in coarse control sediments than fine (Fig 2), although ANOVA and post hoc testing revealed no significant difference between fine and coarse control sediment TOU rates at either 15°C or 20°C (F = 3.873, df = 3, p = 0.22 & p = 0.15 respectively). Model output revealed that both 0.1% and 1% added OM treatments significantly increased logged oxygen uptake rates compared to that of control samples (t = 18.4 and 20.1, p<0.0001 respectively). Furthermore, TOU rates in 20°C incubations were significantly higher than those at 15°C (t = 4.13, p<0.001). Notably, an interaction between coarse sediment fractions at 20°C displayed greater uptake rates than that of fine sediment (t = -2.21, p<0.05). TOU rates across treatments were highly variable with the greatest oxygen flux rates of 112.7 (±17.4) mmol m^-2^ day^-1^ found in coarse sediments with 1% added OM at 20°C. Ambient temperature treatments yielded lower flux rates than that of summer maximum exposures with coarse sediment fractions again exhibiting the higher flux rates (1% coarse 61.1(±10.1) mmol m^-2^ day^-1^, 1% fine 45.2 (±8.69) mmol m^-2^ day^-1^). Control sediments TOU rates varied only slightly between 0.84–2.20 mmol m^-2^ day^-1^ throughout all temperature and sediment grain sizes.

### 3.3. Seawater parameters in VORTs

Oxygen concentration of seawater in ambient temperature and summer maximum temperature VORTs were maintained throughout the study at states of super saturation (10.4 (± 0.0396) mg/L vs 9.57(± 0.0148) mg/L respectively). Ambient temperatures fluctuated with environmental conditions but no significant difference was detected between VORTs. Summer maximum temperature treatments remained steady throughout the study period (20.0°C ± 0.00658). There was no significant difference in pH or salinity of VORT water between or within temperature treatments.

### 3.4. Burial tolerance of *Mytilus edulis*

**Fig 3:**
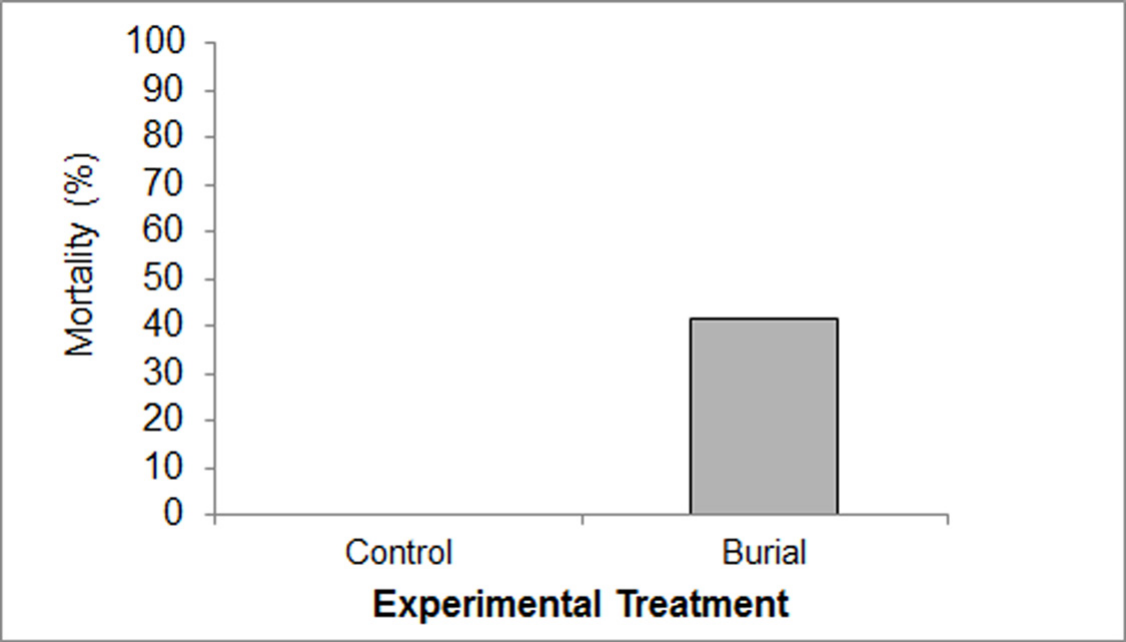
Comparative Mortality of Control and Buried Specimens. Total mortality of buried mussels = 41.7% (all OM, sediment, burial duration and temperature treatments) versus no mortality in controls.

No mussels escaped burial and total mortality of buried individuals was 41.7% (75/180) with no loss of control specimens (with or without chambers) for any duration (Fig 3). Chi-squared analysis revealed a highly significant difference in observed mortality compared with expected values between the control and burial groups (χ^2^ = 66.7, df = 1, p<0.001), therefore controls were omitted from further analysis regarding burial mortality. Remaining data for burial survival was fitted to a GLM with a binomial distribution. The original variables (OM content, temperature, sediment fraction, burial duration (logged to account for uneven time steps in duration treatments), mussel size, VORT number, position in VoRT) were fitted to a saturated logistic regression model. All-possible-subset model selection reduced important predictor variables down to temperature, OM content, sediment fraction and logged burial duration. Interaction terms were introduced between these 4 variables and the model selection process was repeated. Marginal testing Analysis of Variance (F-test) on the reduced best fit model show that sediment fraction size (F = 13.4, p<0.001), logged duration of burial (F = 116.8, p<0.0001), temperature (F = 14.5, p<0.001) and the interaction of OM content with sediment fraction size (F = 12.1, p<0.01) are highly significant predictors of variability within burial mortality data. Determination of absolute model fit yielded an R^2^ value of 0.763 indicating good fit to mortality data.

**Fig 4:**
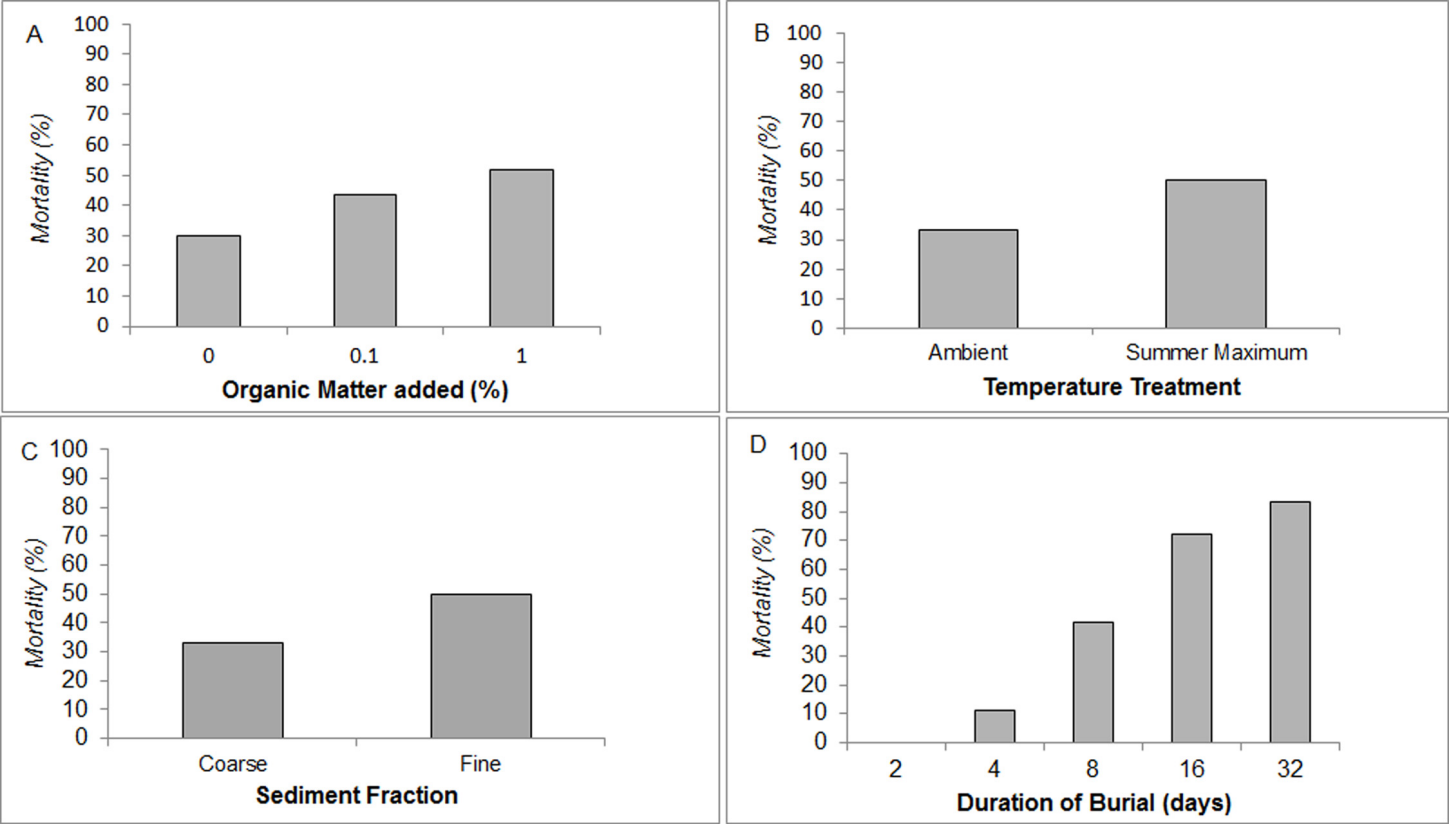
Burial Tolerance under Experimental Variables. Cross tabulation of burial mortality data from **A** organic matter content, **B** temperature, **C** sediment fraction size and **D** duration of burial treatments.

Cross tabulation of pooled mortality data revealed that greater mortality occurred in the 0.1% (43.3%) and 1% (51.7%) OM treatments when compared to 0% OM burial controls (30%). Mortality was observed after 2 days burial duration, with the majority of mortality after 16 days (72.2% and 83.3% mortality at 16 and 32 day burial respectively). Fine sediments yielded greater mortality (50%) than the coarse sediment group (33%). Burials in summer maximum temperature treatments also produced greater mortality (50%) than those incubated at ambient temperatures (33.3%) (Fig 4).

Using the model output, significant changes in probability of mortality were compared with each factor variable’s baseline included in the model’s intercept coefficient. The intercept only model describes the probability of mortality under burial in control sediments, at ambient temperatures in the coarse sediment fraction size within the first two days of burial. Baselines for interactions terms between variables are also included in the intercept only model.

**Fig 5:**
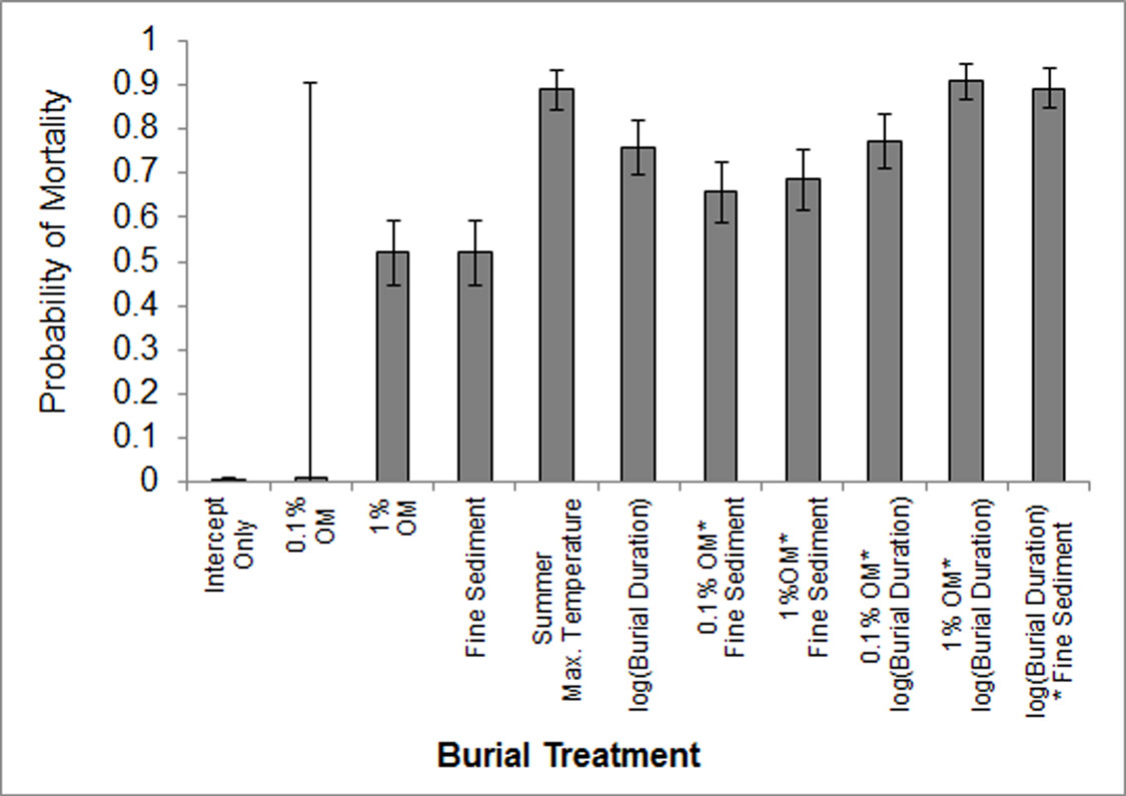
Probability of Mortality under Model Output. Comparison of mortality probability under all model predictor variables compared to probability at the intercept (baseline for all factor variables and ≤ 2 days burial duration). Error bars represent 95% confidence intervals.

Analyses using a hypothesis Z-test revealed that probability of mussel mortality under burial is likely to increase within 2 days under a variety of changes to the ‘intercept only’ model conditions (15°C, 0% OM added, coarse sediment, < 2 days burial). Mortality probability rises significantly from 0.16% (intercept only baseline) when exposed to 1% OM concentrations (52.0%, Z = 13.9, p < 0.0001), fine sediment (52.0%, Z = 13.9, p < 0.0001), summer maximum temperatures (89.1%, Z = 37.9, p < 0.0001), any concentration of added OM in combination with fine sediment (65.7%, Z = 18.5, p < 0.0001 with 0.1% OM and 68.5%, Z = 19.7, p < 0.0001 with 1% OM) or any concentration of added OM over a longer period than 2 days (77.4%, Z = 24.7, P <0.0001 with 0.1% OM and 90.8%, Z = 41.7, p < 0.0001 with 1% OM). Furthermore, burial durations greater than 2 days significantly heighten the probability of mortality from that of the intercept model to 75.8% (Z = 23.6, p < 0.0001). Addition of 0.1% OM to burial mediums represented in the intercept model has no significant effect on survivorship unless considered in interaction with burial duration as above. This is summarised in Fig 5.

## Discussion

The presence of labile organic material in sediment dramatically decreased the burial tolerance of *M*.*edulis* when compared to those smothered in control sediments. Mortality under burial was significantly increased after 2 days in sediments with only 0.1% OM added and the effects of organic enrichment were exacerbated under burial in fine sediment fractions. Furthermore, mortality was significantly increased under burial when incubated by water temperatures close to the likely summer maximum of *M*. *edulis* (20°C) compared to those in ambient (15°C) water.

No mussels were able to escape from burial which corroborates the work of Hutchison *et al*. [18] that *M*. *edulis* can only exhibit this escape behaviour under minor sedimentation events (≤2 cm depth). The ability of the mussel to escape from light smothering but no deeper, may well be a function of metabolic reserves. Considering the species ability to allow oxygen to diffuse into its mantle tissue during aerial exposure [63], it is possible that only under a thin layer of sediment, *M*. *edulis* is able to extract limited oxygen from surrounding pore water. Interstitial water may be relatively oxic under turbulent conditions, permitting at least limited aerobic respiration to continue, although this may be highly reduced in fine sediments with low porosity. In contrast, heavy burial events are likely to rapidly decrease oxygen tensions surrounding the bivalve, prompting the mussel to switch to anaerobiosis [64,65]. Given its prominence in intertidal locations, *M*. *edulis* is robustly tolerant of prolonged anoxia [63] and it is therefore unsurprising that it has shown intermediate tolerance to short term burial events (≤ 32 days) at a variety of burial depths [18]. However it is perhaps logical that the exhibited escape behaviour is impossible during anaerobic metabolism that may be rapidly induced from a severe smothering event. Glycogen is inefficiently degraded anaerobically, with 1 mol yielding only 3 mol of ATP using pyruvate as an electron acceptor compared with full oxidation which yields 37 mol of ATP. Hence the ‘Pasteur effect’ dictates that supporting locomotory activity under anoxic conditions dramatically increases the rate of carbohydrate utilisation, meaning only burst rather than sustained movement is possible [63]. Anaerobic ATP yield may be typically higher in *M*. *edulis* which does not use the standard Embden-Mayerhof-Parnas Pathway [63], instead exploiting a more efficient fermentation process terminating in the accumulation of succinate, alanine and propionate rather than lactate [63,66]. However it is still doubtful that this is sufficient to supply the ATP demand required for escape from burial.

If *M*. *edulis* rapidly switches to anaerobiosis under an inescapable burial event, it is improbable that the liberation of sulphides from anoxic mineralisation of organic material would have immediate implications for mortality (given their inhibitory action upon *aerobic* processes). Sulphide production was confirmed in this study by the presence of sulphide oxidising bacterial mats at the sediment-water interface (*Beggiatoa spp*. with signs of purple sulphur proteobacteria) and the blackening of burial sediments throughout the chambers over time, most likely caused by the production of metal sulphides [45]. However, the presence of these bacterial mats on the surface of burials was not a reliable indicator of mortality. Many specimens under burial in sediment heavily fouled with *Beggiatoa sp*. were still alive upon excavation, indicating their resilience to sulphidic conditions. Previous studies have revealed that bivalves can demonstrate high tolerance to sulphide exposure for relatively short periods [32] and work has shown that blocking the formation of biotic sulphide has no effect on the anoxic survival of a number of bivalve species [67]. Therefore it is unlikely that the significant increases in mortality after just two days in organically charged sediments when compared to controls can be explained through sulphide toxicity.

Bacterial infection may have a more significant role to play on anoxic mussel survival than has been considered during previous burial experiments. Several different bivalve species have exhibited increased susceptibility to pathogenic infection under anoxic conditions [68]. De Zwaan *et al*. [45] found that the anoxic survival time of *Mytilus galloprovincialis* increased with the presence of cadmium and the antibiotic, Chloramphenicol (both of which yield a bacteriostatic effect) in the surrounding medium, even in combination with exogenously added sulphide. The route of infection during anoxic incubation in many bivalves is thought to originate from commensal pathogens that are associated with the bivalve itself [42,44] and a variety of external factors including increases in salinity, pH and temperature may exacerbate bacterial proliferation [43]. The presence of nitrogen rich substrates such as the fish pellets used in this study may provide the ideal environment for bacterial communities that rely on the degradation of peptides or subsequent products of proteolysis for metabolism [43]. Also, it should be mentioned that neither sediments nor fish pellets were anti-bacterially treated prior to burials. Therefore it is possible that external rather than commensal pathogens were introduced to the mussels and a potential source of mortality. Method of infection of the mussels is beyond the scope of this paper but sterilisation of the burial medium may help future studies discover the route of transfer. Importantly, protocol described here may provide a closer approximation to in-situ conditions where homogenised biota and marine sediment are themselves a sink for microbial communities [69].

Given the ability of *M*. *edulis* to survive in a highly reducing, sulphidic environment, the presence of a potential source and ideal substrate for bacterial growth in combination with the highly advanced stage of decomposition in dead mussels under organically charged sediments, it is suggested that the introduction of labile organic matter into burial mediums may increase mortality of buried mussels compared with controls through the facilitation of pathogenic infection. This may also help explain the significant increases in buried mussel mortality under summer maximum treatments.

The effect of temperature on bacterial populations is of significance in organic carbon mineralisation. Diagenesis of marine organic matter can be coarsely divided into two main phases that are conducted by a complex community of bacteria. The first stage of hydrolysis converts complex macromolecules into smaller molecules that may easily permeate bacterial membranes for further decomposition. From here the terminal phase of mineralisation consists of organic carbon oxidation by other distinct bacterial communities using a variety of electron acceptors including oxygen or sulphate [70]. Although the activation energy needed for the oxidation of organic carbon may be variable throughout different sediment conditions, increased temperature raises the susceptibility of organic material to initial microbial attack by attaining the minimal temperature for fermentative microbial processes to occur. It is attaining this critical activation energy that is thought to be the limiting factor in determining the rapidity of net community response [70–72]. Once the initial response is achieved, high temperatures will also elevate microbial catalytic performance, increasing the overall rate of organic matter metabolism [73].

Thus thermal facilitation is demonstrated by the oxygen uptake experiments. Assuming oxygen flux as a proxy for organic decomposition, mineralisation rates were dramatically increased under higher temperatures. The 1% organic loadings in coarse and fine sediments reached a mean TOU rate of 112.7 (±17.4) mmol m^-2^ day^-1^ and 71.4 (±18.2) mmol m^-2^ day^-1^ respectively at 20°C compared with 61.1 (±10.1) and 45.2 (±8.67) mmol m^-2^ day^-1^ at 15°C. Speeding the initial bacterial response and metabolism of reactive organic matter and increasing the abundance of proteolytic end products, rapidly provides the ideal sere for succession for sulphate reducing bacteria (SRB) such as *Desulphobulbus spp*.[43]. These conditions may also be crucial to the multiplication of additional pathogenic communities [43]. While the proliferation of these pathogens was not monitored for this study, the incidence of sulphide oxidising bacteria on the surface of burials occurred earlier at high temperatures, suggesting an advanced rate of SRB community activation and so it is conceivable that this also applied to pathogenic strains.

While the increase in bacterial activity caused by the presence of organic material may well be exacerbated under higher temperatures, it is likely that this is not the sole cause of increased mortality under summer maximum conditions. Many marine invertebrates rely on poikilothermic metabolism, their body temperature closely matching that of the external environment [74]. Hence increased temperatures will cause a general rise in the metabolic rate of mussels [75] by increasing catalytic performance of enzyme controlled metabolic conversions 1–3 fold for every rise of 10°C [76]. In order to maintain homeostatic control during seasonal temperature fluctuations, mussels have demonstrated the ability to chronically down-regulate their metabolism during times of thermal stress, with a notable drop in amplitude of the respiration response at the start of summer [77]. However, sharp fluctuations in temperature that may be experienced in shallow sub-tidal habitats during the summer could rapidly increase metabolic rates beyond a point at which fermentative processes in smothered mussel populations could sustain the ATP demand. This may become crucial in affected communities That said, resilience of *Mytilus spp*. to these acute variations in thermal stress may be higher in individuals associated with warmer habitats [78,79] and so temperature sensitivity under burial may be dependent upon thermal history. Consequently, we recognise that there may be differences in survivorship under temperature fluctuations between cultured mussels used in this study and wild stock, as demonstrated in other species [80].

Notably, burial mortality in this study was enhanced under fine sediments in combination with 1% added organic content. This may be explained by sediment dynamics demonstrated in the TOU experiments. Although temperature was a key determinant in sediment metabolism, TOU was also increased with coarser, high porosity sediment. For example, coarse 0.1% OM sediment treatments displayed a greater mean oxygen flux rate at 20°C than that of fine, 1% treatments at the same temperature (98.4 and 71.4 mmol m^-2^ day^-1^ respectively). Overall organically loaded coarse sediment fractions displayed surprisingly high uptake rates at both experimental concentrations even in the low temperature group (61.1 (±10.1) mmol m^-2^ day^-1^ at 1% OM and 36.5 (±4.26) mmol m^-2^ day^-1^ at 0.1%). As a reference, typical TOU values for heavily fouled sediments associated with aquaculture pens may fall in the range of 150–450 mmol m^-2^ day^-1^ [25,81]. Yet rates were approximately a third lower under fine sediment.

The reasons for this are most likely two fold. Firstly the relative contribution of sulphate reduction to TOU from the oxidation of sulphides at the DBL has been estimated as high as 50–100% [82] and sediments with larger grain sizes have a greater diffusion capacity for the transfer of solutes to the water column [83]. This leads to increased re-oxidation of sulphides in the water column [84] thus altering oxygen uptake rates. This is consistent with the observation of earlier colonisation of *Beggiatoa spp*. on the surface of coarse experimental sediments at either temperature. Secondly, increased permeability of the coarse aggregates may increase advective oxygen supply through the sediment and allow aerobic sediment metabolism at centimetre scale depths [85]. Further support is offered by significant differences in TOU detected between fraction sizes in sediments only where OM was added (i.e. not in controls), suggesting that inflated TOU rates from coarse sediment with 0.1% and 1% reactive OM added are an artefact of increased advective processes in a more porous medium.

The differences in organic content detected between fine and coarse sediments during LOI analyses are of little concern to sediment biogeochemistry. Firstly, the calculation of organic content using LOI analysis in organically poor sediments is likely to overestimate OM values due to pyrolysis of inorganic compounds such as carbonates [86], despite efforts to minimise this through the use of lower temperatures (500°C) compared with other published protocols [87]. Secondly, the assumption that these kiln dried sediments would be of approximately equal lability is supported by lack of any detectable difference in TOU rates between fine or coarse control sediment. Thus each aggregate fraction size were treated equally when adding organic material.

Increased advection through permeable aggregates could also explain increased survival of *M*. *edulis* under coarse sediment. It is possible that further penetration of oxygen would prevent the maintenance of the anoxic environment needed for the establishment of anaerobic pathogens discussed earlier. Indeed it has been shown that finer sediments are more resistant to fluctuations in pore water conditions, allowing stability in bacterial populations [88]. To a small extent, increased aerated flushing under porous aggregate [85] may also increase the incidence of oxygen diffusion into the mussel mantle cavity, increasing ATP yield from aerobic processes. While this is likely to be of low importance in organically loaded sediments with high oxygen flux rates, it could explain the reduced mortality of control burials of *M*. *edulis* under large grain sizes and the highest mortality under the combination of 1% organic matter and fine sediment.

In summary, it is clear that the presence of reactive organic matter within sediment has a significant influence upon the survival of *M*. *edulis* under burial. The decomposition of this labile material quickly utilises oxygen in sediment pore space through decomposition and maintains a highly reducing environment. This, in combination with providing a nitrogen rich substrate for bacterial introduction and growth, most likely maintains optimum conditions for the propagation of pathogenic microbes. It is probable that this process is facilitated with high organic loadings combined with fine sediment where interstitial conditions are most stable (supported by the strong interaction between fine sediment and organic loading in our model). Increased temperature to the limits of thermal tolerance dramatically increases the probability of mortality of buried mussels, with a combination of metabolic stress on *M*. *edulis* specimens and increased microbial action likely explanatory variables. While the down-regulation of metabolic rates before summer may alleviate seasonality of risk, acute changes in temperature may impose metabolic demands that cannot be met through burial-induced anaerobiosis. This could have profound implications for *M*. *edulis* beds in shallow sub-tidal zones affected by aggregate outwash. Disregarding considerations of the role of organic matter during these over-sanding events could lead to an underestimation of the impact from aggregate outwash upon the survival of benthic fauna.

What must be considered is that community responses to sedimentation events will be highly dependent on faunal composition and environmental conditions at the site of extraction and aggregate overburden. Organisms accustomed to high energy, sedimentary environments are likely to be far more tolerant of over-sanding events than those residing in less mobile substrate [89,90]. Invertebrate mobility and ability to escape burial is a major factor affecting resilience to these perturbations [16,18]. Moreover, while *M*. *edulis* has displayed short-term tolerance to sulphidic conditions, organic enrichment of dredged areas has the potential to significantly hinder benthic recovery and re-colonisation by other species with a highly reducing environment [91]

While what are discussed are hypothesised explanations of the distal effect of OM in burial sediments, further work should aim to address proximate causation mechanisms that are beyond the scope of this paper. Physico-chemical monitoring of the burial medium along with characterising bacterial community change would provide a more thorough explanation of sediment dynamics. Additionally the introduction of an antibacterial agent prior to burial may be a good measure of the influence of pathogenic communities.

Expansion on this study should also aim to consider processes of bioturbation that may occur during over-sanding events, the biochemical influence of natural benthic sediment onto which organically loaded discards may fall or the influence of colder temperatures which were not investigated due to the seasonality of this work. This considered, the effect of the temperatures used in this study provide worst case scenario data for UK waters, allowing any management strategies that use this information to employ the precautionary principle in respect to the effect of smothering on mussel beds.

Finally it does not escape our attention that other anthropogenic activities such as deep sea mining and the burgeoning marine renewable industry will probably create similar environmental impacts to marine aggregate extraction. The data presented here in this paper therefore have the potential generic application wherever a mix of organic and inorganic sedimentation is likely.

## Supporting Information

S1 Table Summary of Burial Treatments. Details of replicate numbers for all combinations of added organic matter to burial mediums, burial duration, incubation temperature and sediment grain size.
(TIF)

S2 Table Summary of Control Treatments. Details of replicate numbers for all combinations of unburied (potted or un-potted) controls under different incubation duration, and temperatures.
(TIF)
