# Supplementary figures and images for: The Influence of Organic Material and Temperature on the Burial Tolerance of the Blue Mussel, Mytilus edulis: Considerations for the Management of Marine Aggregate Dredging
Source: PLoS One. 2016 Jan 25;11(1):e0147534. doi: 10.1371/journal.pone.0147534 (PMC4726446; doi:10.1371/journal.pone.0147534)

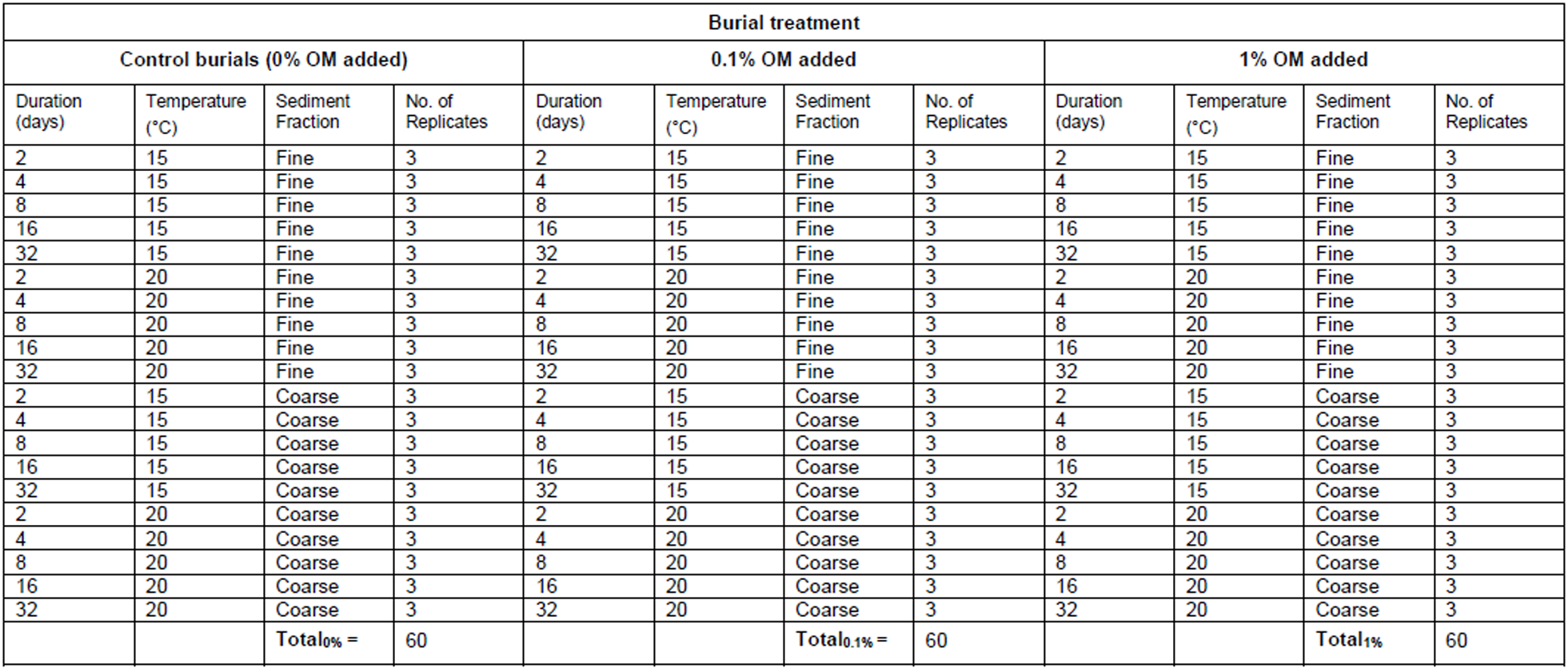

Supplement: S1 Table — Details of replicate numbers for all combinations of added organic matter to burial mediums, burial duration, incubation temperature and sediment grain size. (TIF) [file pone.0147534.s001.tif]

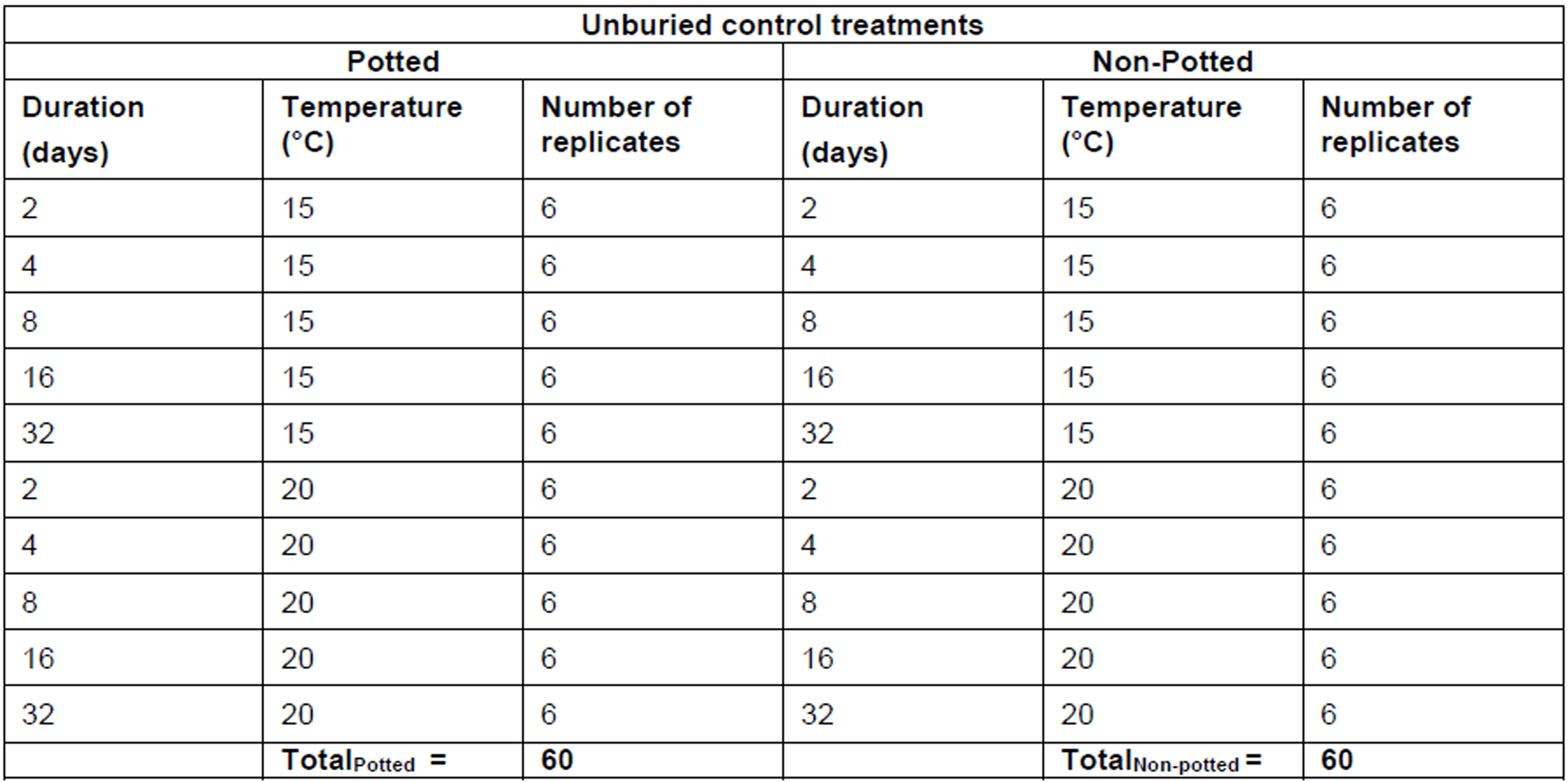

Supplement: S2 Table — Details of replicate numbers for all combinations of unburied (potted or un-potted) controls under different incubation duration, and temperatures. (TIF) [file pone.0147534.s002.tif]
